# In Vivo Analysis of the Dynamic Motion Stability Characteristics of Geese’s Neck

**DOI:** 10.3390/biomimetics7040160

**Published:** 2022-10-12

**Authors:** Jiajia Wang, Haoxuan Sun, Wenfeng Jia, Fu Zhang, Zhihui Qian, Xiahua Cui, Lei Ren, Luquan Ren

**Affiliations:** 1 College of Agricultural Equipment Engineering, Henan University of Science and Technology, Luoyang 471003, China; 2 Key Laboratory of Bionic Engineering (Ministry of Education), Jilin University, Changchun 130022, China

**Keywords:** goose’s neck, motion stabilization, biplane X-ray

## Abstract

The goose’s neck is an excellent stabilizing organ with its graceful neck curves and flexible movements. However, the stabilizing mechanism of the goose’s neck remains unclear. This study adopts a dynamic in vivo experimental method to obtain continuous and accurate stable motion characteristics of the goose’s cervical vertebra. Firstly, the results showed that when the body of a goose was separately moved back and forth along the Y direction (front and back) and Z direction (up and down), the goose’s neck can significantly stabilize the head. Then, because of the limitation of the X-ray imaging area, the three-dimensional intervertebral rotational displacements for vertebrae C4–C8 were obtained, and the role that these five segments play in the stabilization of the bird’s neck was analyzed. This study reveals that the largest range of the adjacent vertebral rotational movement is around the *X*-axis, the second is around the *Y*-axis, and the smallest is around the *Z*-axis. This kinematic feature is accord with the kinematic feature of the saddle joint, which allows the flexion/around *X*-axis and lateral bending/around *Y*-axis, and prevents axial rotation/around *Z*-axis.

## 1. Introduction

The necks of birds are highly flexible and highly mobile, which effectively compensates for the bird’s inability to turn its eyes and improves the flexibility of the head when feeding, drinking, and performing other tasks [1]. In addition, the high flexibility of the bird’s neck provides the basis for head stability during ground movements and flights [2,3,4]. In some birds, the head is relatively stationary in space through the “holding state” of the “head nod” movement when walking [5,6,7], and the ability to gaze steadily when their bodies move back and forth [8] suggests that the neck has some stabilizing function. Revealing the mechanism behind this unique movement pattern of birds’ necks could inspire new ideas with scientific value and practical significance for developing engineering technologies towards stability maintenance, anti-shaking, vibration damping, and vibration isolation.

The unique structural features of the cervical vertebrae of birds are the basis for maintaining the stable movement of their heads. The bird’s neck usually consists of 13–25 vertebrae [9], with 14–15 vertebrae being more typical and the joint being saddle-shaped [10]. The unique joint structure of the bird’s neck allows for a significant degree of movement in dorsoventral flexion and extension and lateral flexion, but is limited in axial rotational movement [11]. Studies on the morphological characteristics of the avian neck have focused on skeletal muscles [12,13,14,15] and genetic evolution. Krings et al. used X-ray and CT scanning techniques to simultaneously acquire 3D head movements and 3D 14-segment cervical vertebrae (C1–C14) models of American barn owls to quantify the morphological characteristics of individual vertebrae, including cervical spinal canal diameter and articular protrusion, intervertebral joint parameters including joint center distance, and pitch angle [16]. Terray et al. described nine modular cervical vertebrae that are common among different bird species by performing 3D surface structural morphometry on 187 cervical vertebrae collected from 16 bird species [17].

The dorsiflexion, ventral flexion, lateral flexion movements, and rotational movements of the neck of pigeons, domestic chickens, turkeys, geese, owls, and other birds were mainly studied through cadaveric experiments and modeling simulations [2,18,19,20,21,22,23]. Furet et al. obtained a 3D model of the vertebrae by CT scanning and developed a system of equations between the vertebrae models. A constraint was then defined to estimate the range of motion of the vertebrae [22]. Some researchers studied the way animal spines moved by analyzing X-ray data from the animals. X-ray fluoroscopy was used to obtain the natural posture of the neck during head rotation in live and cadaveric owls, and the shape of a single vertebra was obtained by CT images. The results showed that the rotation motion could be described as a combination of motion in the yawing and rolling axes by Krings et al. [23]. Kambic et al. studied the range of motion (RoM) of the three-dimensional cervical joints along the cranio-caudal axis in wild turkey carcasses, and grouped the RoM of the avian neck into three regions. The cranial joints primarily perform ventral flexion and extension with a high degree of axial rotational mobility and lateral flexion and extension. The caudal joint is predominantly dorsiflexed and has low axial rotational mobility with high lateral flexion mobility. The axial rotational mobility of the middle joint is variable and exhibits low lateral flexion [2].

The above studies analyzed the cervical spine motion mechanism of different birds through static cadaver experiments and modeling simulations, which showed that the cervical spine of birds mainly performed dorsiflexion and ventral flexion movements, with higher lateral flexion activity and lower axial rotation activity of the vertebrae. The cadaver experiments can obtain accurate intervertebral motion; however, they are mainly static studies, and the posture is artificially placed. Therefore, dynamic in vivo studies are needed in order to fully and sufficiently reveal the natural motion of bird cervical vertebrae.

This study adopted a dynamic in vivo experimental method to obtain continuous and accurate natural motion characteristics of the goose’s cervical vertebra. When the body of a goose was separately moved back and forth along the Y-direction and Z-direction, the three-dimensional intervertebral rotational displacements of the C4–C8 vertebrae of the goose could be obtained by the in vivo dynamic measurement method. Furthermore, the motion characteristics of the cervical vertebra were analyzed, aimed at explaining the stabilizing mechanism of the bird’s neck.

## 2. Materials and Methods

### 2.1. Cervical Spine 3D Structure Construction and Coordinate System Establishment

In this study, a moderately sized adult goose weighing 4.1 kg was selected as the experimental subject. CT images of the cervical spine skeleton of the goose were acquired prior to the biplane X-ray test, and the scans were acquired on Somatom Definition AS (Siemens, Munich, Germany) with the parameters provided by the supplemental files. After the CT medical image data of the goose’s neck was acquired, the Dicom format file was exported, and reverse modeling was performed using Mimics (Version17.0, Materialize, Leuven, Belgium) for later 2D-3D alignment of biplane 3D dynamic X-ray motion data.

**Figure 1:**
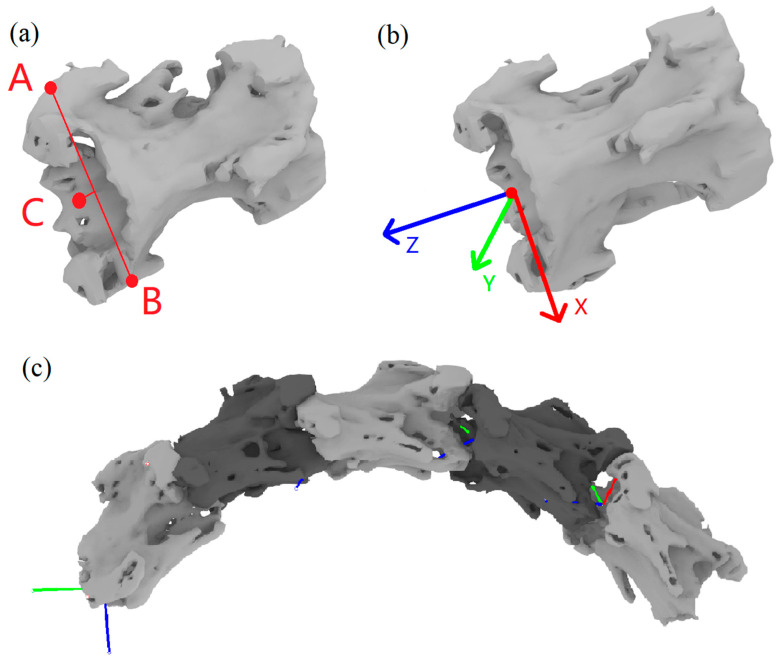
C4–C8 cervical spine modeling and coordinate system establishment: (**a**) Positioning points and auxiliary lines of the vertebrae: A/B—the most lateral point on the two anterior articular processes, C—the ventral midline point of the vertebral foramen on the cranially terminated articular surface of the vertebra; (**b**) diagram of vertebral coordinate system; (**c**) vertebrae combination diagram.

An anatomical coordinate system of the goose cervical spine was established for measuring joint motion, as shown in Figure 1. Two vectors were used to create 3D axes for the cranially terminated articular surfaces of each vertebra: a horizontal vector by manually identifying the most lateral point on the two anterior articular processes; and an axial vector by manually identifying the ventral midline point of the vertebral foramen on the cranially terminated articular surface of the vertebra. The vertical vector is calculated by intersecting the horizontal vector with the axial vector, and then the axial vector was intersected with the vertical vector to create a 3D coordinate axis. These three vectors defined the anatomical coordinate system of each vertebra [24]. The coordinate systems on each pair of adjacent vertebrae were used to calculate the relative rotational motion in follow-up X-ray experiments.

### 2.2. Biplane X-ray Image Acquisition

**Figure 2:**
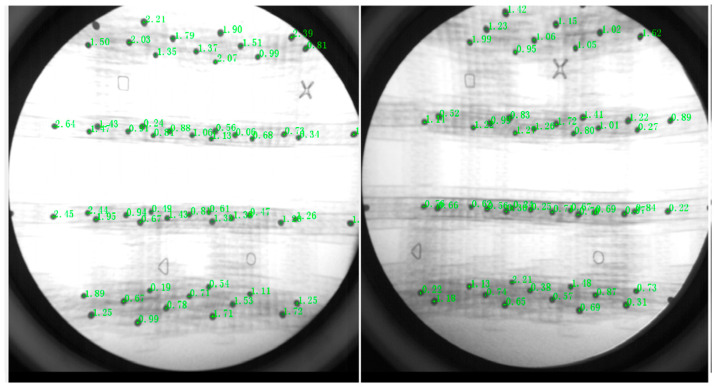
Systematic error correction.

To calibrate the biplane X-ray data, the X-ray image data of the calibrator cube and the calibrator patch were acquired in this study [25]. Before the 2D-3D alignment of the biplane X-ray data, the data calibration of the images of the calibrator cube and calibrator patch acquired above needed to be performed in the XMALab software. The X-ray data of the acquired calibrator cube and patch as well as the csv and ref files of the calibrator cube were imported into the XMALab software, while the reference points (square, circle, triangle and cross) of the calibrator cube under the calibration module Calibration are clicked sequentially and the test error is calculated automatically, as shown in Figure 2.

**Figure 3:**
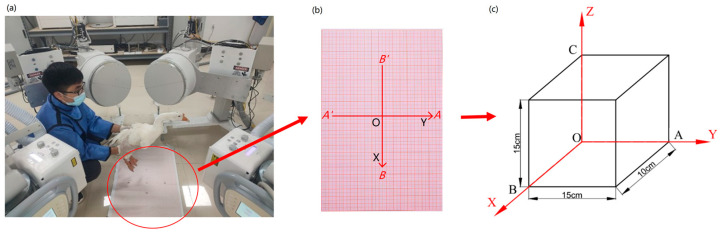
Schematic diagram showing the direction of the biplane X-ray test: (**a**) Test process (**b**) Coordinate system in the test (**c**) Coordinate system and direction: O-Center point, OA-Displacement in the Y direction/front and back, OB-Displacement in the X direction/left and right, OC-Displacement in the Z direction/up and down. (Rotation around the *X*-axis is forward flexion and dorsiflexion, rotation around the *Y*-axis is lateral flexion, and rotation around the *Z*-axis is axial rotation).

The motion measurement auxiliary system was set as shown in Figure 3. First of all, the coordinate paper was placed on the ground, and the starting position and the end position of the goose motion range was marked. The experimental moving range of the goose along the *X*-axis, *Y*-axis, and *Z*-axis was ±10 cm, ±15 cm, and ±15 cm, respectively. The body of the goose was moved from the initial position/zero point to the maximum positive position and back to the zero point, then moved from the zero point to the maximum negative position and back to the zero point. Each round of the test was repeated six times for a total of 18 tests. During the experiment, only the body of the goose is moved, and the head and neck of the goose are not disturbed by external forces in order to simulate the geese’s natural movement posture. The goose’s neck motion data was acquired using a biplane X-ray motion capture system (ISSI, Milpitas, CA, USA). The parameters of the biplane X-ray equipment at the time of acquisition were: sampling frequency of 100 fps, exposure time of 1000 us, a voltage of 50 kV, a current of 80 mA, and other parameters were set as the default values of the system.

### 2.3. Biplane X-ray Data Processing

**Figure 4:**
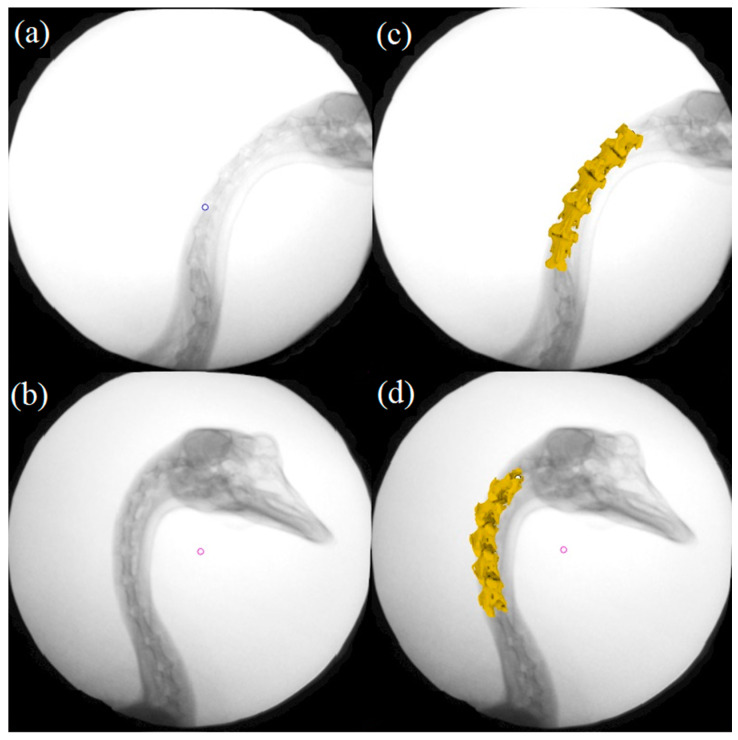
Relationship between model and radiograph positions before and after alignment: (**a**,**b**) X-ray images before alignment, (**c**,**d**) X-ray images after alignment.

Because of the limited imaging scope of the X-ray equipment, the overall motion of the goose neck could not be captured completely, so the focus of this study was on the fourth to eighth consecutive cervical vertebrae (C4–C8) of the goose. The anatomical coordinate system was established at the cranial end joint surfaces of these five vertebrae, respectively. Subsequently, the six degrees of freedom joint data was obtained by comparing the translation and rotation elements of the anatomical coordinate system of different vertebrae in Rhino 6.0 (Robert McNeel & Assoc, Seattle, WA, USA). By setting up an X-ray virtual environment in the Rhino software, the photographed cervical joint model can be translated and rotated in 3D space, simulating the experimental process of projecting X-rays onto the joint and imaging it in the receiving plane, enabling a virtual image of the joint to be obtained. This image was analyzed by edge comparison with the actual photographed joint image. After iteration and optimization, the best match between the unique virtual image and the real joint image could be calculated in 3D. This process is called 2D-3D alignment, as shown in Figure 4.

By aligning and measuring the anatomical coordinate system of the adjacent vertebrae to obtain the exact three-dimensional spatial position of the goose cervical joint during the X-ray test, a series of continuous angular motion data of the goose cervical joint can be obtained. The spatial rotation angle of each joint was analyzed to investigate the mechanism of stability maintenance in the goose neck.

## 3. Results

**Figure 5:**
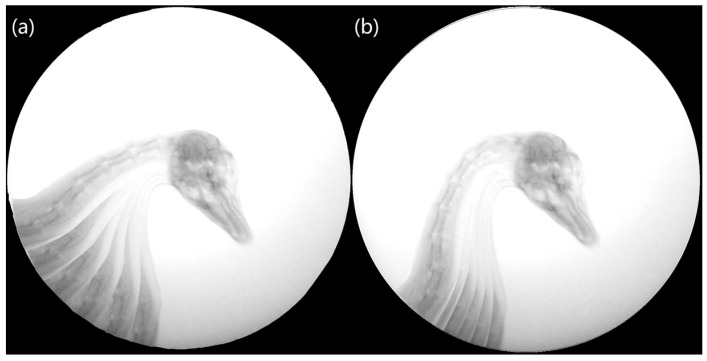
Motion synthesis diagram in X-rays: (**a**) Goose moving in Y-direction, (**b**) Goose moving in Z-direction.

The X-ray experimental image series was obtained for the described three movement directions. The results showed that the goose’s neck can carry out various motion styles freely. As shown in Figure 5, the stability of the goose’s head was observed obviously for two trails inaccessibly: when moving the body in the Y-direction and the Z-direction. Therefore, the further study about the motion stability analysis was focused on these two representative trails in the study. In each trail, the goose’s cervical intervertebral continuous angular displacement of C4/C5, C5/C6, C6/C7, and C7/C8 were obtained.

### 3.1. Stability Analysis of Goose’s Neck When Moving the Body in Y-Direction

**Figure 6:**
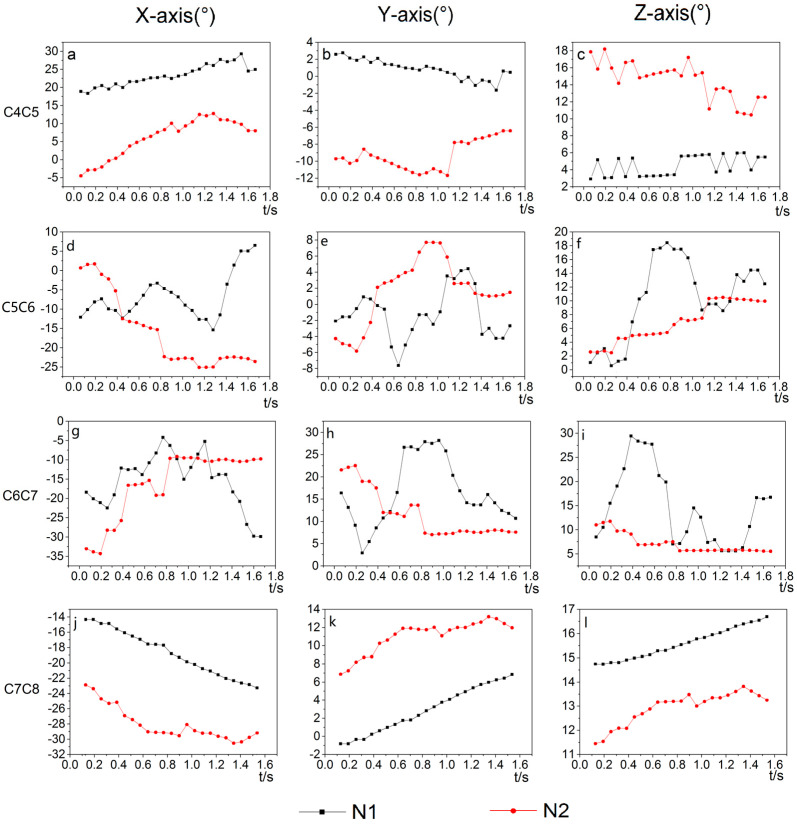
The angular three-dimensional displacements of joints C4/C5, C5/C6, C6/C7, and C7/C8 for two representative trails (N1, N2): (**a–c**) angle of C4/C5 around X, Y and Z axes, (**d–f**) angle of C5/C6 around X, Y and Z axes, (**g–i**) angle of C6/C7 around X, Y and Z axes, (**j–l**) angle of C7/C8 around X, Y and Z axes.

When moving the goose’s body back and forth in the Y-direction, the cervical intervertebral rotation angles of C4/C5, C5/C6, C6/C7, and C7/C8 around the X, Y, and *Z*-axis were obtained. Two representative trails were selected to analyze the stability of the goose’s neck. Figure 6 shows the continuous angular displacement of C4/C5, C5/C6, C6/C7, and C7/C8 joints.

As shown in Figure 6a–c, j–l, the curve trends of N1 and N2 for C4/C5 and C7/C8 joints are roughly similar, while the curve trends of N1 and N2 for C5/C6 and C6/C7 joints show slight differences. In Figure 6d–f, g–i, the N1 and N2 curves of C5/C6 joints and C6/C7 joints fluctuated a lot and have a slight similarity.

The range of motion for each joint around the X, Y, and Z axes are shown in Table 1. The motion ranges of joints C4/C5 and C5/C6 around the *X*-axis are larger, indicating that the forward flexion and dorsiflexion is the predominant motion in these two joints of the bird’s neck, while the lateral flexion and axial rotation were auxiliary motions. The range of motion in the three axes of the joint is not significantly different, with dorsiflexion, lateral flexion, and axial rotation occurring simultaneously. Overall, the rotation along the *X*-axis has a larger range of motion than along the other two axes, and the C5/C6 and C6/C7 joints have a larger range of motion than the other joints. In summary, when moving the body along the Y-direction, the goose exhibited a larger motion range around the *X*-axis, while the motion range around the *Y*-axis and *Z*-axis were relatively less.

### 3.2. Stability Analysis of Goose’s Neck When Moving the Body in Z-Direction

**Figure 7:**
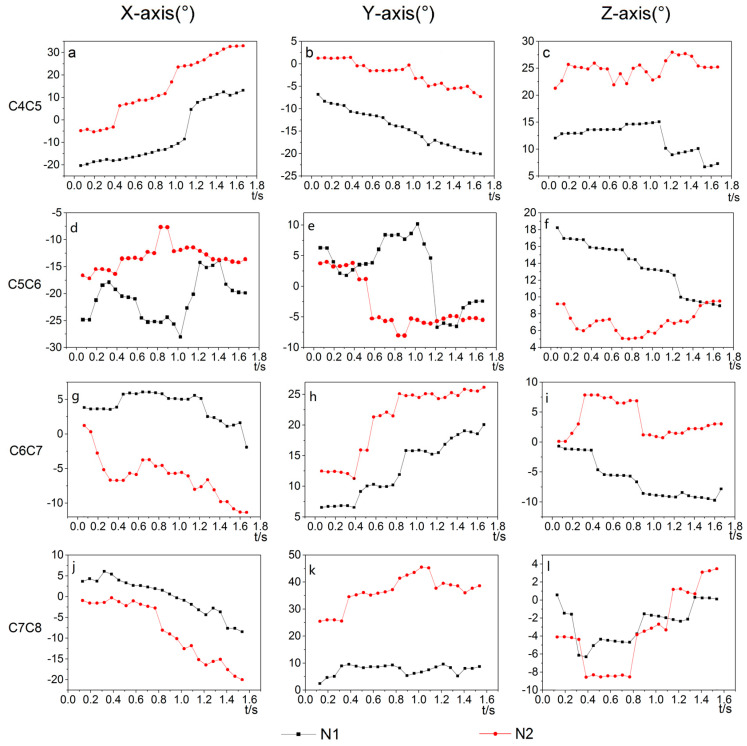
The angular three-dimensional displacement of joints C4/C5, C5/C6, C6/C7, and C7/C8 for two representative trails (N1, N2): (**a–c**) angle of C4/C5 around X, Y and Z axes, (**d–f**) angle of C5/C6 around X, Y and Z axes, (**g–i**) angle of C6/C7 around X, Y and Z axes, (**j–l**) angle of C7/C8 around X, Y and Z axes.

When moving the goose’s body back and forth in the Z-direction, the cervical intervertebral rotation angles of C4/C5, C5/C6, C6/C7, and C7/C8 around the X, Y, and *Z*-axis were obtained. Two representative trails were selected to analyze the stability of the goose’s neck. Figure 7 shows the continuous angular displacement of C4/C5, C5/C6, C6/C7, and C7/C8 joints.

As shown in Figure 7a–c, g–l, the cure trends of N1 and N2 for C4/C5, C6/C7, and C7/C8 joints are roughly similar. In Figure 7d–f, the N1 and N2 curves for the C5/C6 joints show significant differences.

The range of motion of each joint in the X, Y, and Z directions are shown in Table 2. The motion range of joints C4/C5 and C7/C8 around the *X*-axis are larger, indicating that the forward flexion and dorsiflexion are the predominant motion in these two joints of the bird’s neck, while the lateral flexion and axial rotation are auxiliary motions. The motion range of the C5/C6 and C6/C7 joints around *Y*-axis are larger, indicating that the lateral flexion are the predominant motion of these two joints, while the flexion and dorsiflexion and axial rotation were auxiliary motions. Overall, the range of motion along the X and Y axes is larger, and the motion range of joint C4/C5 is larger than the other joints. In summary, when moving the body along the Z-direction, the goose exhibited a larger motion range around the *X*-axis and the *Y*-axis, and the *Z*-axis was relatively less.

In this paper, using a biplanar three-dimensional dynamic X-ray motion capture system, the passive dimensional stability motion data of the goose’s neck in the body subjected to the Y-direction and Z-direction were collected to obtain the corner data of the adjacent joints of C4–C8 of the goose cervical spine. The test shows that by analyzing the range of motion of the C4–C8 joints of the goose neck vertebrae, it was found that the goose neck is significantly moved around the *X*-axis and *Y*-axis.

## 4. Discussion

This study verified the feasibility of combining CT scanning and a biplane X-ray motion capture system for the analysis of the neck movement scheme of geese. Compared with the traditional methods such as scanning animal carcasses by X-ray and capturing motion data by single-plane X-ray, the motion data captured by this method is more continuous and natural, which provides a more effective scheme for the subsequent study of an animal in vivo motion mechanism.

In this study, when moving the body along the Y-direction and Z-direction, the angular data of the C4/C5, C5/C6, C6/C7, and C7/C8 joints of the goose cervical spine were analyzed and compared. It was found that the range of motion of the goose cervical joints was the largest in the direction of anterior flexion and dorsiflexion, the second largest in the range of lateral flexion, and the smallest in the range of axial rotation. This result was in accord with the previous study [11]. This phenomenon is likely caused by the kinematic feature of the saddle joint, which allows the flexion/around the *X*-axis and the lateral bending/around the *Y*-axis, and prevents axial rotation/around the *Z*-axis. Limited by the acquisition range of the biplane X-ray equipment, the study focused on the clear C4–C8 portion of the goose neck motion in the study. By surveying the range of motion of the joints, it can be found that the C4/C5 joints had a larger range of motion, and the other joints gradually decreased. This is consistent with the description by Kambic et al that C4/C5 was more flexible. [2]. The three-dimensional stabilization motion of the goose neck when moving the goose body in two typical motion directions was analyzed, and it can be seen that the vertebrae of the goose neck performed anterior/dorsal flexion, lateral flexion, and axial rotation simultaneously. We propose that the complex joint morphology provides an explanation for the coupled lateral flexion and axial rotation, which was put forward by Kambic et al. [2].

At present, only the skeletal structure analysis and skeletal movement pattern analysis have been conducted in this study, and the role of the nerves and muscles in the neck of the goose in stable movement needs to be studied. In the future, the existing studies on skeletal structure and movement patterns could be combined to complete the study on the mechanism of a goose’s neck movement characteristics.

## Figures and Tables

**Table 1:** Data of each joint movement when moving the body in Y-direction.

Joint	Test Number	Coordinate Axes	Movement Data	
			Range of Motion	Interval Size
C4C5	N1	X	18.337°~29.262°	10.925°
		Y	−1.639°~2.746°	4.385°
		Z	2.91°~5.98°	3.07°
	N2	X	−4.482°~12.778°	17.26°
		Y	−11.667°~−6.406°	5.261°
		Z	10.46°~18.19°	7.73°
C5C6	N1	X	15.438°~6.482°	21.92°
		Y	−7.632°~4.413°	12.045°
		Z	0.575°~18.421°	17.85°
	N2	X	−25.115°~1.731°	26.85°
		Y	−5.823°~7.701°	13.524°
		Z	2.46°~10.49°	8.03°
C6C7	N1	X	−29.916°~−4.211°	25.705°
		Y	2.897°~28.16°	25.263°
		Z	5.581°~29.447°	23.867°
	N2	X	−34.271°~−9.146°	25.125°
		Y	7.024°~22.543°	15.519°
		Z	5.55°~11.754°	6.203°
C7C8	N1	X	−23.28°~−14.336°	8.95°
		Y	−0.83°~6.823°	7.657°
		Z	14.738°~16.693°	1.955°
	N2	X	−30.54°~−22.881°	7.65°
		Y	6.854°~13.183°	6.329°
		Z	11.451°~13.819°	2.368°

**Table 2:** Data of each joint movement when moving the body in Z-direction.

Joint	Test Number	Coordinate Axes	Movement Data	
			Range of Motion	Interval Size
C4C5	N1	X	−20.374°~13.117°	33.491°
		Y	−20.068°~−6.821°	13.246°
		Z	6.694°~15.07°	8.376°
	N2	X	−5.316°~32.974°	38.289°
		Y	−7.314°~1.411°	8.725°
		Z	21.293°~27.98°	6.687°
C5C6	N1	X	−28°~−13.852°	14.152°
		Y	−6.716°~10.203°	16.92°
		Z	8.932°~18.209°	9.276°
	N2	X	−17.187°~−7.694°	9.49°
		Y	−8.064°~3.983°	12.047°
		Z	5.026°~9.507°	4.481°
C6C7	N1	X	−1.915°~6.064°	7.979°
		Y	6.531°~20.064°	13.532°
		Z	−9.737°~−0.729°	9°
	N2	X	−11.347°~1.21°	12.558°
		Y	11.268°~26.148°	14.88°
		Z	0.111°~7.853°	7.74°
C7C8	N1	X	−8.5°~6.056°	14.556°
		Y	2.431°~9.581°	7.15°
		Z	−6.304°~0.566°	6.87°
	N2	X	−20.019°~−0.276°	19.743°
		Y	25.438°~45.51°	20.072°
		Z	−8.56°~3.46°	12.02°

## Data Availability

Not applicable.

## Supplementary Materials

The following supporting information can be downloaded at: https://www.mdpi.com/article/10.3390/biomimetics7040160/s1, Table S1: CT scan parameters table.
