# Supplementary material for: In Vivo Analysis of the Dynamic Motion Stability Characteristics of Geese’s Neck
Source: Biomimetics (Basel). 2022 Oct 12;7(4):160. doi: 10.3390/biomimetics7040160 (PMC9590001; doi:10.3390/biomimetics7040160)
Supplement: Supplementary file 1 [file biomimetics-07-00160-s001.zip › biomimetics-1935705-supplementary.pdf]

CT scan parameters table

**Scan parameters**	**numeric**
Effective	150mAs
Voltage	80kv
Slice	1.0mm
Scan time	11.09s
Rotation time	0.5s
Delay	2s
